# H_2_O_2_ Signalling Pathway: A Possible Bridge between Insulin Receptor and Mitochondria

**DOI:** 10.2174/157015912804143559

**Published:** 2012-12

**Authors:** Igor A Pomytkin

**Affiliations:** Biosignal Ltd., Moscow, Russia

**Keywords:** Alzheimer’s disease, brain, insulin receptor, hydrogen peroxide, mitochondria.

## Abstract

This review is focused on the mechanistic aspects of the insulin-induced H_2_O_2_ signalling pathway in neurons and the molecules affecting it, which act as risk factors for developing central insulin resistance. Insulin-induced H_2_O_2_ promotes insulin receptor activation and the mitochondria act as the insulin-sensitive H_2_O_2 _source, providing a direct molecular link between mitochondrial dysfunction and irregular insulin receptor activation. In this view, the accumulation of dysfunctional mitochondria during chronological ageing and Alzheimer’s disease (AD) is a risk factor that may contribute to the development of dysfunctional cerebral insulin receptor signalling and insulin resistance. Due to the high significance of insulin-induced H_2_O_2_ for insulin receptor activation, oxidative stress-induced upregulation of antioxidant enzymes, e.g., in AD brains, may represent another risk factor contributing to the development of insulin resistance. As insulin-induced H_2_O_2_ signalling requires fully functional mitochondria, pharmacological strategies based on activating mitochondria biogenesis in the brain are central to the treatment of diseases associated with dysfunctional insulin receptor signalling in this organ.

## INTRODUCTION

Hydrogen peroxide (H_2_O_2_) and superoxide anion radicals, collectively known as reactive oxygen species (ROS), are traditionally regarded as toxic byproducts of aerobic metabolism. Therefore, evidence that insulin action is facilitated by ROS was considered a redox paradox [1]. Most knowledge in this area comes from studies in fat cells. As early as the 1970s, insulin was discovered to stimulate the production of intracellular H_2_O_2_ in adipose tissue [2, 3]. Later, it was found that a membrane-bound NADPH oxidase, catalysing the reduction of molecular oxygen to superoxide, produced ROS in fat cells in response to insulin stimulation [4-6]. ROS are involved in upregulating very early insulin receptor signalling, since inhibition of the insulin-induced ROS generation attenuates tyrosine phosphorylation of the insulin receptor and its substrates [7, 8]. Protein tyrosine phosphatases (PTP), which dephosphorylate and inactivate insulin receptors, are a target of insulin-induced ROS [7-9]. H_2_O_2_ is a particularly important ROS for insulin receptor signalling, given that catalases almost completely inhibit the effects of insulin-induced ROS [8]. Thus, there is a signalling pathway enhancing very early insulin receptor signalling in fat cells, which involves NADPH oxidase-mediated H_2_O_2_ generation. 

Emerging evidence suggests that mitochondria are the insulin-sensitive source of ROS essential for insulin receptor activation in neurons. These findings provide a direct molecular link between mitochondrial function and insulin receptor signalling, highlighting a role of mitochondrial dysfunction in the development of insulin resistance. This review is focused on the mechanistic aspects of the insulin-induced H_2_O_2_ signalling pathway in neurons and the factors affecting sit, which could promote the development of insulin resistance. 

## INSULIN RECEPTOR IS REGULATED BY TYROSINE PHOSPHORYLATION

Cerebral insulin is produced by the pancreas and enters the cerebrospinal fluid by receptor-mediated saturable transport [10]. Little or no insulin is produced in the brain itself. Insulin binds to its cognate receptors and elicits a variety of biological responses [11]. Mice lacking the insulin receptor gene *via *targeted disruption die within the first week of birth due to severe diabetic ketoacidosis [12, 13]. Most knowledge regarding insulin receptor signalling comes from studies in classical insulin target tissues such as fat, muscle and the liver, where insulin is essential for regulating energy functions such as glucose and lipid metabolism. Unlike its peripheral counterpart, the central insulin receptor is involved in anorexigenic responses, fertility and reproduction, memory formation, and neuronal survival, and is considered to have no direct effect on neuronal glucose metabolism [14-20].

The insulin receptor is a heterotetrameric protein composed of two α-subunits and two β-subunits linked by disulphide bonds [11]. The extracellular α-subunits have sites for insulin binding. The intracellular portion of the transmembrane β-subunits contains the insulin-regulated tyrosine kinase. The neuron-specific isoform of the insulin receptor (isoform A) differs from the peripheral insulin receptor (isoform B) and arises from the alternative splicing of exon 11, which removes 12 amino acids near the COOH terminus of the α-subunit [21, 22]. There are some notable functional distinctions between the neuron-specific receptor and its peripheral counterpart. The neuronal insulin receptor binds insulin with a two-fold higher affinity, can also bind insulin-like growth factor-II with physiologically relevant affinity, shows no negative cooperativity, and has increased rates of receptor internalisation [23-26]. The neuronal isoform is the predominant insulin receptor isoform in the brain, with the highest density in the olfactory bulb, cerebral cortex, hypothalamus and hippocampus, where it is concentrated at synapses as components of postsynaptic densities [27-29]. 

The reversible tyrosine phosphorylation of the insulin receptor lies at the core of insulin signalling. Upon insulin binding, the insulin receptor undergoes autophosphorylation at three critical tyrosines within the activation loop of the tyrosine kinase domain, whose modification dramatically increases tyrosine kinase activity and triggers the downstream signalling cascades [30-32]. 

Dephosphorylation of the insulin receptor is catalysed by several members of the protein tyrosine phosphatase family, including the intracellular PTP1B, which has a major physiological role in the negative regulation of the insulin receptor itself and downstream effectors [9, 33-35]. There is evidence that a decrease in PTP1B activity improves insulin receptor signalling in neurons. A decrease in hypothalamic PTP1B lowers food intake, reducing body weight and improving insulin action and signalling in the hypothalamus [36]. A conditional deletion of PTP1B in the retina was shown to enhance insulin receptor signalling and cell survival [37]. Mice lacking PTP1B demonstrate increased tyrosine phosphorylation of the insulin receptor, improved systemic insulin sensitivity and obesity resistance [38, 39]. 

It is generally accepted that PTP1B is a physiologically relevant target of insulin-induced H_2_O_2_ [1]. A catalytic cysteine at amino acid position 215 of PTP1B is highly susceptible to direct oxidation by micromolar H_2_O_2_ [40-42]. Depending on the reaction conditions, PTP1B undergoes a variety of inhibitory post-translational modifications, of which the S-glutathionylated form seems to be predominant in the intracellular milieu where glutathione is abundant [43-45]. The inhibitory S-glutathionylation is reversible and PTP1B can be reactivated by the glutaredoxin and thioredoxin systems [42]. As PTP1B dephosphorylates the already active phosphorylated form of the insulin receptor, H_2_O_2_-mediated PTP1B inhibition regulates insulin receptor activity in cells. 

## INSULIN-INDUCED ROS ARE REQUIRED FOR INSULIN RECEPTOR TYROSINE PHOSPHORYLATION IN NEURONS

**Fig. (2):**
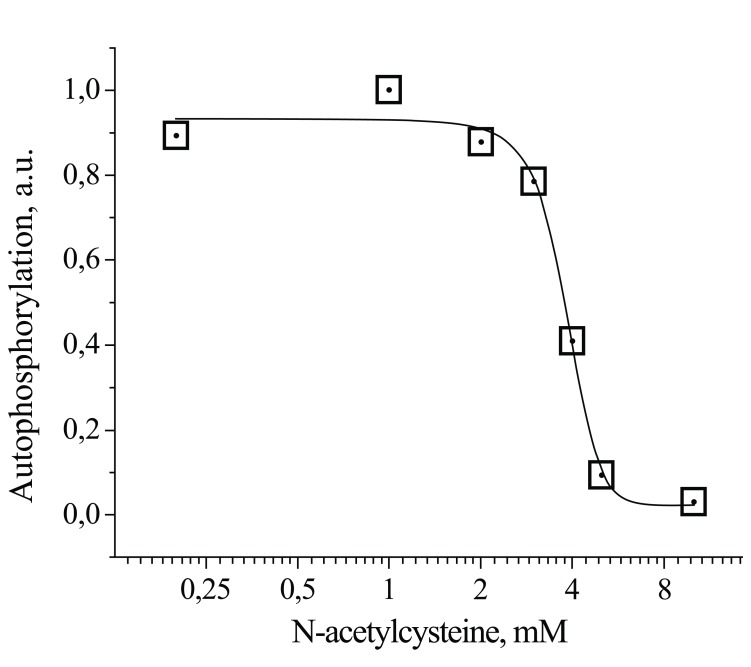
Dependence of insulin-stimulated insulin receptor autophosphorylation on NAC concentrations in CGN cultures. CGN were exposed to insulin 100 nM and increasing NAC concentrations. Receptor autophosphorylation was assayed as described in [53].

Some evidence suggests that insulin-stimulated H_2_O_2_ plays a critical role in early insulin receptor signalling in neuronal cells. Human neuroblastoma SK-N-BE(2) cells, which do not produce ROS in response to insulin, are insulin insensitive and demonstrate a lack of tyrosine phosphorylation of their insulin receptor and its substrates upon insulin stimulation [46-48]. Insulin stimulation of primary cerebellar granule neurons (CGN) was shown to trigger insulin receptor autophosphorylation, which was accompanied by the immediate release of H_2_O_2_ [53]. Fig. (**1**) shows that the duration of the insulin-induced H_2_O_2_ spike in CGN was less than 30 s. The average quantity of hydrogen peroxide per spike per µg of protein was 2.5 ± 0.2 nM, a one-order of magnitude greater than the baseline H_2_O_2_ production in non-stimulated neurons (0.20 ± 0.01 nM; p < 0.05). This insulin-induced H_2_O_2_ was shown to be important for the activation of insulin receptors, since the H_2_O_2_ scavenger N-acetylcysteine (NAC) completely abolished insulin receptor autophosphorylation in CGN (Fig. **2**). 

Two sources of insulin-induced H_2_O_2 _in cells have been identified to date, a membrane-bound NADPH oxidase in adipocytes [4-6] and mitochondrion in tissues rich in mitochondria (liver, heart and neurons). In neuronal tissue, insulin-stimulated H_2_O_2_ production has been shown to be sensitive to selective inhibitors of mitochondrial ROS production (malonate and FCCP) [53] as well as to diphenyleneiodonium (DPI) [49], a non-selective inhibitor of mitochondrial H_2_O_2 _production during reverse electron transport at mitochondrial complex II [50-52]. It should be noted that although DPI is frequently used as an inhibitor of stimulus-induced H_2_O_2_ production, it does not discriminate between mitochondria and NADPH oxidases as sources of H_2_O_2_ production, since DPI is a non-selective inhibitor of both. 

Given that insulin-stimulated insulin receptor autophosphorylation is sensitive to inhibitors of mitochondrial ROS production, the process of mitochondrial superoxide/H_2_O_2_ generation is briefly summarised below. 

## MITOCHONDRIA AS H_2_O_2_ SOURCE

Mitochondria are considered to be the main quantitative source of superoxide anion radical and H_2_O_2_ in mammalian cells [54]. The superoxide is a primary mitochondrial ROS, which undergoes rapid stoichiometric dismutation to H_2_O_2_ in the presence of cytoplasmic (SOD1) or mitochondrial (SOD2) superoxide dismutase. Since the rate of non-enzymatic dismutation of superoxide to H_2_O_2 _is three orders of magnitude lower than the enzymatic one, SOD1 and SOD2 are critical components of signalling pathways where H_2_O_2_ is a secondary messenger.

It is generally accepted that much of the superoxide generated by these organelles results from leakage of electrons in two components of the mitochondrial electron transport chain: complex I (NADH-ubiquinone oxidoreductase) and complex III (ubiquinol-cytochrome c oxidoreductase) [55]. Complex I provides the highest rates of superoxide generation in isolated mitochondria from the brain, heart and muscle during the reverse electron flow from succinate-ubiquinone oxidoreductase (complex II). Rates of superoxide generation at complex III are considerably lower [52, 56-59]. Complex I releases superoxide into the mitochondrial matrix, where it is transformed stoichiometrically into H_2_O_2_ by superoxide dismutase SOD2 (Mn-SOD) [54, 58-61]. As the reactivity of MnSOD is high (rate constant of 2 x 10^9^ M^-1^ s^-1^) and its concentration in the mitochondria is 10 µM, the steady-state concentration of mitochondrial superoxide is very low [62, 63]. Although superoxide anion radical itself cannot diffuse through the mitochondrial inner membrane, being a charged molecule at physiological pH, its neutral metabolite H_2_O_2 _readily passes through the mitochondrial membranes into the cytoplasm. Therefore, the major signalling form of mitochondrial ROS is H_2_O_2_.

The majority of mitochondrial superoxide is produced by complex I during reverse electron transport (RET). RET occurs in the presence of a significant proton-motive force, when electron flow from complex II gives electrons to coenzyme Q, which, in turn, gives electrons to complex I to produce the superoxide. Mitochondria respiring on the complex II substrate succinate display the highest rates of RET-associated H_2_O_2_ production [56, 57, 64]. This H_2_O_2_ generation is extremely sensitive to changes in the proton-motive force. When respiration is coupled to ATP synthesis, mitochondria produce very low amounts of H_2_O_2_. However, even a small increase in the proton-motive force above a threshold value slightly exceeding the value in the active (phosphorylating) state gives rise to a very steep increase in H_2_O_2_ production [56, 65, 66]. Depletion of the proton-motive force by the addition of uncouplers, inhibitors or ADP completely abolishes H_2_O_2_ generation [56, 57, 67]. 

Several lines of evidence suggest that the RET-associated H_2_O_2_ production in isolated mitochondria is regulated by the activity of complex II. During state 4 respiration, when complex II has the highest succinate dehydrogenase activity [68], the rate of H_2_O_2_ generation is also the highest [56, 65]. In the transition to state 3, when complex II is rapidly deactivated [59], H_2_O_2_ generation is abolished [56, 65]. Factors deactivating complex II (e.g., uncouplers, inhibitors and ADP) also inhibit RET-associated H_2_O_2_ generation [68]. Malonate, the classic complex II inhibitor, dose-dependently slows the rates of RET and rotenone-sensitive superoxide production at complex I [69]. Succinate, the complex II substrate, promotes superoxide production at complex I [70]. The rate of H_2_O_2_ production in isolated mitochondria depends on succinate concentrations and is in excellent compliance with Michaelis-Menten kinetics, meaning that the succinate dehydrogenase reaction is the rate-limiting step in RET-associated H_2_O_2_ generation [71]. In view of all this evidence, complex II seems to be a key regulatory point, which controls rates of mitochondrial H_2_O_2_ production through varying the rate of succinate oxidation. 

Complex II activity is tightly regulated by a reversible oxidation-reduction reaction. Oxaloacetate (OAA), an intermediate of the citric acid cycle and an inhibitor of succinate dehydrogenase [72], binds to the oxidised form of complex II with an affinity at least one order of magnitude greater than to the reduced form [73]. Therefore, complex II is inhibited by oxaloacetate upon oxidation and becomes activated upon reduction, when it liberates oxaloacetate [73]. Consequently, the enzyme is obtained largely in the deactivated form, containing tightly bound oxaloacetate in a 1:1 ratio to the enzyme [74]. Ubiquinol, a reduced form of coenzyme Q, is a physiologically relevant reductant and activator of complex II [75, 76]. The highest succinate dehydrogenase activity is observed in state 4 of respiration, when coenzyme Q is largely in the ubiquinol form and capable of reactivating complex II [58]. In the transition to state 3, when the ubiquinol pool is depleted *via *oxidation in the Q cycle, the rapid oxidative deactivation of complex II occurs [58, 75]. Micromolar H_2_O_2 _reversibly inhibits complex II activity by enhancing oxaloacetate-mediated inactivation of complex II [77, 78]. This inhibition has been observed in intact mitochondria [77, 78] and synaptosomes [79].

As a summary, the succinate dehydrogenase reaction plays a key role in mitochondrial superoxide/H_2_O_2_ formation. There is a switch-like dependence of the rate of succinate-supported H_2_O_2 _generation on the mitochondrial membrane potential and even a small decrease in the potential below the threshold value completely abolishes H_2_O_2 _release by the mitochondria. 

## MITOCHONDRIA AS SOURCE OF INSULIN-INDUCED H_2_O_2_

Several lines of evidence suggest that the mode by which insulin stimulates H_2_O_2_ generation in mitochondrion-rich tissues is by varying the rate of succinate oxidation. Insulin stimulation triggers almost immediate H_2_O_2_ release in the liver, heart and neurons, which is completely inhibited by malonate, a classic mitochondrial complex II inhibitor [53, 80]. A dramatic transient increase in the rates of succinate-supported H_2_O_2_ generation was observed in mitochondria isolated from tissues (liver and heart) pre-treated for 1 min with physiological insulin concentrations, as compared to mitochondria isolated from non-stimulated tissues (control) [71]. In terms of Michaelis-Menten kinetics, the insulin pretreatment resulted in a 3- to 4-fold increase in Vmax and a 2- to 4-fold decrease in Km to a value of 2 to 9 µM succinate. For reference, succinate levels in human plasma vary from 1 to 9 µM at rest and increase to up to 125 µM in hypoxic conditions [81, 82]. Therefore, a significant increase in insulin-induced H_2_O_2_ generation can be achieved under steady-state physiological succinate levels through the acute increase in the rate of succinate oxidation at complex II. This is in full agreement with previous data related to insulin-stimulated oxidation of the isotope-labelled [2,3-^14^C] and [1,4-^14^C] succinates in rat liver and muscle cells, where insulin stimulation acutely increased only the rates of mitochondrial [2,3-^14^C]succinate oxidation, which was observed as early as within 30 s [83].

Mitochondria are an insulin-sensitive H_2_O_2_ source involved in insulin-stimulated receptor autophosphorylation in neurons [53]. Malonate, the specific inhibitor of mitochondrial complex II, inhibits H_2_O_2_ release and insulin receptor autophosphorylation in neurons stimulated with insulin. Succinate, a substrate of complex II, enhances insulin receptor autophosphorylation stimulated with suboptimal insulin concentrations. The uncoupler FCCP, a mitochondrion-depolarising agent and inhibitor of mitochondrial ROS production, inhibits insulin-induced H_2_O_2_ release and insulin receptor autophosphorylation. Hence, insulin-induced H_2_O_2_ generation in neurons is succinate-dependent and sensitive to uncoupler-induced mitochondrial depolarisation.

Altogether, these findings suggest that insulin stimulates the well-known process of succinate-dependent mitochondrial H_2_O_2_ production, while components transducing the signal between the insulin receptor and mitochondria remain to be elucidated. 

Fig. (**3**) illustrates three modes of operation of complex II. In mode 1, when mitochondria actively produce ATP and the ubiquinol-to-ubiquinone ratio UQH_2_/UQ is low, complex II is inhibited by binding with oxaloacetate and the mitochondria does not produce H_2_O_2_ or does but at low rates. In mode 2, when there is no ATP production and there is a high ubiquinol-to-ubiquinone ratio and high proton-motive force, complex II is de-inhibited and gives electrons to complex I to produce the superoxide. The superoxide undergoes a dismutation reaction with mitochondrial Mn-SOD to produce H_2_O_2_, which passes through the mitochondrial membranes into the cytoplasm. In this mode, mitochondria produce H_2_O_2_ continuously at high rates. In mode 3, upon insulin stimulation, complex II becomes activated and provides succinate oxidation at rates of about one-order of magnitude higher than that in mode 2. In this mode, the mitochondria produce H_2_O_2_ at the highest rates, but for a short time (seconds).

**Fig. (4):**
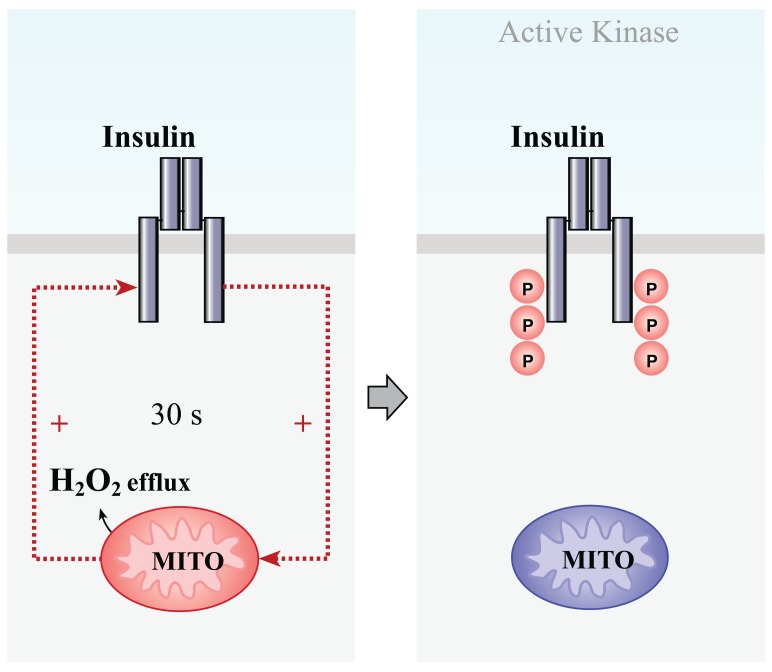
Scheme of the cooperation between mitochondria and the insulin receptor during insulin receptor activation in neurons.

It is possible to draw some tentative conclusions regarding a mode of cooperation between insulin receptor and mitochondria during insulin receptor activation (Fig. **4**). 

Insulin binding to the cognate receptor stimulates almost the immediate release of an H_2_O_2_ spike from the mitochondria, which, in turn, upregulates autophosphorylation of the insulin receptor and activation of the receptor tyrosine kinase. This double positive regulatory feedback loop seems to involve the H_2_O_2_-mediated inhibition of protein tyrosine phosphatases, which otherwise dephosphorylate the insulin receptor and, thus, prevent full activation of the insulin receptor tyrosine kinase. Upon activation, the insulin receptor kinase triggers downstream signalling. Therefore, the insulin-induced H_2_O_2_ signalling pathway plays a role in enhancing very early insulin receptor signalling.

The unexpectedly high dependence of insulin receptor autophosphorylation (i.e., activation) on mitochondrial H_2_O_2_ in neurons suggests that alterations in the mitochondrial machinery of H_2_O_2_ production may disrupt insulin receptor activation. In this view, the possible relationships between mitochondrial dysfunction and insulin receptor signalling and between insulin-induced H_2_O_2_ and oxidative stress are discussed below.

## RELATIONSHIPS BETWEEN INSULIN-INDICED H_2_O_2_ AND MITOCHONDRIAL DYSFUNCTION

Since mitochondrial H_2_O_2_ is required for insulin receptor activation, it is obvious that some alterations in mitochondrial function could result in a malfunction in insulin receptor activation and insulin resistance. Requirements that must be met to trigger insulin-induced H_2_O_2_ signalling include a high proton-motive force, a high ubiquinol-to-ubiquinone ratio and a complete conversion of ADP to ATP. Thus, it is clear that only fully functional mitochondria may serve as the insulin-sensitive source of H_2_O_2_ and support insulin receptor activation. This provides a link between mitochondrial dysfunction and insulin resistance that is highly prevalent in ageing and Alzheimer’s disease (AD). 

Chronological ageing is accompanied by a progressive decline in brain mitochondrial functions such as respiration with the complex I substrate NADH, enzymatic activity of complex I and complex IV, and ATP production [117]. Along with this, brain mitochondria are chronically depolarised in senescence [118, 119]. Since age is the major risk factor for AD, the aging process itself plays an important role in promoting mitochondrial dysfunction in the brain. Indeed, mitochondrial dysfunction is one of the earliest and most prominent features in sporadic age-associated AD, as has been highlighted in excellent reviews [120-122]. Therefore, the age-related decline in mitochondrial functions seems to disrupt insulin receptor activation in neurons and lead to the development of cerebral insulin resistance in old age. 

## RELATIONSHIPS BETWEEN INSULIN-INDUCED H_2_O_2_ AND OXIDATIVE STRESS

Oxidative stress is defined as an imbalance between oxidants and antioxidants in favour of the oxidants, potentially leading to damage [84]. Insulin-stimulated H_2_O_2_ generation, which is low and short-term, does not contribute to significant oxidative damage in cells. As the threshold level for H_2_O_2_ neurotoxicity in cerebellar granule neurons is 10 µM [85], the insulin-induced H_2_O_2_ production of about 100 nM [53] is too low to induce apoptosis. The reaction of H_2_O_2_ with ferrous iron (Fe^2+^), which generates hydroxyl radicals suspected of being involved in protein carbonylation, lipid peroxidation and DNA oxidation [86], is too slow to occur, compared to the much higher rates of the competitive reactions of H_2_O_2_ removal *via *the peroxiredoxin-thioredoxin system [87]. Indeed, a calculated half-time for the degradation of 100 nM H_2_O_2_ with ferrous iron is hours, when the iron brain concentrations are as high as 300 µM [88] and the second-order rate constant is 4400 M^-1^ s^-1^ [89]. Therefore, insulin-stimulated H_2_O_2_ production is far too low to contribute to oxidative damage in neurons. 

On the contrary, there is evidence suggesting that oxidative stress may directly affect insulin receptor signalling. Micromolar H_2_O_2_ inhibits insulin receptor autophosphorylation [90, 91], insulin binding to the receptor, insulin-induced tyrosine phosphorylation of the receptor substrates and downstream signalling [90]. 

## RELATIONSHIPS BETWEEN INSULIN-INDUCED H_2_O_2_ AND ANTIOXIDANT SYSTEMS

Insulin-induced H_2_O_2_ signalling is downregulated by intracellular antioxidants. There are several antioxidant systems that have been shown to be effective in scavenging intracellular H_2_O_2_. Among them, the peroxiredoxin-thioredoxin and glutathione peroxidase-glutathione systems are the most important on the basis of competitive kinetic analysis. Peroxiredoxins (Prx) and glutathione peroxidase (Gpx1) were revealed to be the primary H_2_O_2_ scavengers responsible for the majority of hydrogen peroxide metabolism, according to kinetic estimations based on published rate constants and abundance data [92, 93]. Gpx1 is a selenoprotein that catalyses the reaction of glutathione and H_2_O_2_ at a second-order rate constant of 6 x 10^7^ M^-1^ s^-1^ [92]. The oxidised glutathione is then reduced by NADPH with glutathione reductase to complete the cycle. Peroxiredoxin family members (Prx1 through to Prx6) are thiol-dependent non-catalytic peroxidases that scavenge H_2_O_2_ directly at a second-order rate constant of about 10^7^ M^-1^ s^-1^ [94]. The oxidised peroxiredoxin is reduced by thioredoxin, which is in turn reduced by NADPH-thioredoxin reductase. Therefore, the glutathione peroxidase-glutathione and peroxiredoxin-thioredoxin systems are channels that transfer reducing equivalents from NADPH to H_2_O_2_ with the highest efficacy compared to other cellular antioxidant systems. The replenishment of the NADPH pool depends basically on the activity of glucose-6-phosphate dehydrogenase, a rate-limiting enzyme in the pentose phosphate pathway, which supplies reducing equivalents to cells [95]. 

Some data suggest strong ultrasensitivity of insulin-induced H_2_O_2_ signalling to the activity of antioxidant systems. Fig. (**2**) shows the dependence of the insulin-stimulated insulin receptor autophosphorylation on pretreatment with N-acetylcysteine (NAC). NAC is a thiol compound, which is widely used for modelling antioxidant effects in *in vitro* experiments. Although the direct non-enzymatic reaction of NAC with H_2_O_2_ is slow [96], NAC is at least three-orders of magnitude more effective in the enzymatic reaction with H_2_O_2_ catalysed by glutathione peroxidase [97]. Thus, NAC is an artificial molecule that mimics glutathione in the glutathione peroxidase reaction. Even a small increase in NAC levels above a threshold value completely prevents insulin-stimulated insulin receptor activation. The curve fitting provides a Hill slope of 7.8, which indicates strong ultrasensitivity of insulin receptor autophosphorylation to intracellular antioxidant activity. Therefore, intracellular antioxidant systems represent an effective barrier for insulin-induced H_2_O_2_ signalling, which is operated in an “all-or-none” mode depending on the antioxidant activity. It can be hypothesised then that excessive activation of antioxidant systems could induce dysfunctional activation of insulin receptors and consequently, insulin resistance. 

Some evidence obtained from animal studies supports this idea. Mice overexpressing glutathione peroxidase, Gpx1, developed insulin resistance associated with hyperinsulinemia, hyperglycemia, obesity and a 70% reduction in insulin-stimulated phosphorylation of insulin receptors, compared to wild type control mice [98]. On the contrary, mice lacking Gpx1 were protected from high-fat diet-induced insulin resistance, while administration of NAC rendered them more insulin-resistant and increased fasting glucose levels in the blood [99]. 

AD is a progressive neurodegenerative disease, which is commonly characterised by irregular cerebral insulin receptor signalling and insulin resistance [100-103] and elevated antioxidant defense, possible to compensate for oxidative stress. Oxidative stress is regarded as a primary progenitor of AD because oxidative markers appear prior to beta-amyloid senile plaques [104-106]. The compensatory upregulation of antioxidant systems has been found in AD. Activity of glucose-6-phosphate dehydrogenase is increased in AD brains as a response to oxidative stress [107-109]. A significant overexpression of glutathione peroxidase has been found in the AD hippocampus [110, 111]. Moreover, several studies have demonstrated the upregulation of peroxiredoxins in AD brains. Protein levels of Prx1 and Prx2 were significantly increased in AD brains than in age-matched control [112]. Prx1 was overexpressed in the human AD cortex [113]. Prx2 was significantly increased in the frontal cortex [114] and hippocampus of AD brains [115]. Prx6 was markedly elevated in astrocytes in many regions of AD brains [116]. Altogether, these findings indicate that oxidative stress provokes a significant increase in the capacity of antioxidant systems to metabolise H_2_O_2_ in AD. In view of the ultrasensitivity of insulin receptor autophosphorylation to antioxidant activity in neurons, the elevated activity of the antioxidant systems in AD may contribute to dysfunctional insulin receptor activation and central insulin resistance. 

## MITOCHONDRIAL BIOGENESIS ACTIVATION AND TREATMENT OF INSULIN RESISTANCE

As insulin-induced H_2_O_2_ signalling requires fully functional mitochondria, pharmacological strategies based on activating mitochondria biogenesis in the brain seems to be central to the treatment of diseases resulting from dysfunctional insulin receptor signalling such as cognitive impairments and AD. Some evidence supports this idea. It has been postulated that peroxisome proliferator-activated receptor-gamma agonists (PPARγ) may enhance cognition in AD patients by improving mitochondrial function [123]. Rosiglitazone, a PPARγ agonist and stimulator of mitochondrial biogenesis in the brain [124], restores insulin responsiveness and rescues behavioural deficits in Tg2576 transgenic mice [125]. In a small clinical study, 6 months of rosiglitazone treatment resulted in enhanced memory and cognitive function in AD patients compared to the placebo-treated control [126]. In a phase II clinical trial, involving over five hundred patients with mild to moderate AD, rosiglitazone treatment enhanced attention and memory, while patients with apolipoprotein E4 allele did not respond to the therapy [127]. 

## CONCLUSIONS

Insulin-induced H_2_O_2_ signalling establishes a bridge between insulin receptors and mitochondrial function in neurons. Although the insulin-induced H_2_O_2_ spike is short-term, it has unexpectedly high significance for the autophosphorylation of insulin receptors, the critical post-translational modification leading to the activation of the receptor tyrosine kinase. The set of requirements that must be met to trigger insulin-induced H_2_O_2_ generation indicates that only fully functional mitochondria may serve as the insulin-sensitive source of H_2_O_2_. It provides a direct molecular link between mitochondrial dysfunction and derangements in insulin receptor signalling and insulin resistance. Therefore, an accumulation of dysfunctional mitochondria during chronological ageing is a risk factor that contributes to the development of age-related diseases known to accompany dysfunctional cerebral insulin receptor signalling, e.g., age-associated cognitive deficits and Alzheimer’s disease.

Compensatory upregulation of antioxidant enzymes under oxidative stress is another risk factor contributing to the development of insulin resistance in view of the high significance of insulin-induced H_2_O_2_ for insulin signalling. As intracellular antioxidant defense under oxidative stress is calibrated against elevated oxidant levels, this higher level of defense becomes a barrier for insulin-induced H_2_O_2_ signalling and prevents insulin receptor activation. Since insulin receptor autophosphorylation is ultrasensitive to changes in the activity of antioxidant systems, the compensatory upregulation of antioxidant enzymes under oxidative stress seems to be a key factor contributing to the development of insulin resistance in neurons. 

As insulin-induced H_2_O_2_ signalling requires fully functional mitochondria, pharmacological strategies based on activating mitochondria biogenesis and/or mitochondria turnover in the brain are central to the treatment of diseases resulting from dysfunctional insulin receptor signalling in this organ. 

## Figures and Tables

**Fig. (1):**
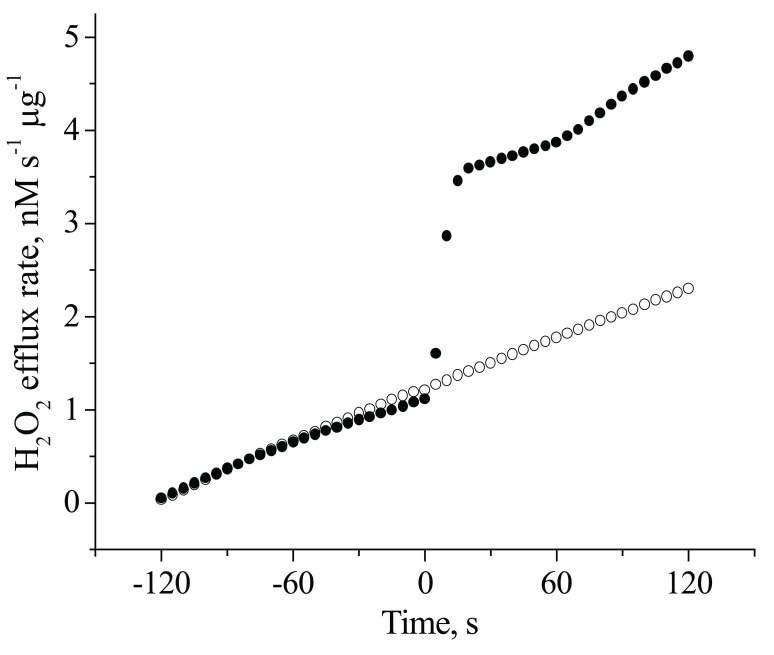
Time-dependence of insulin-induced H_2_O_2_ generation in CGN cultures. CGN were exposed to insulin 100 nM (●) or medium (○) at zero time. H_2_O_2_ release was assayed with Amplex Red as described in [53].

**Fig. (3):**
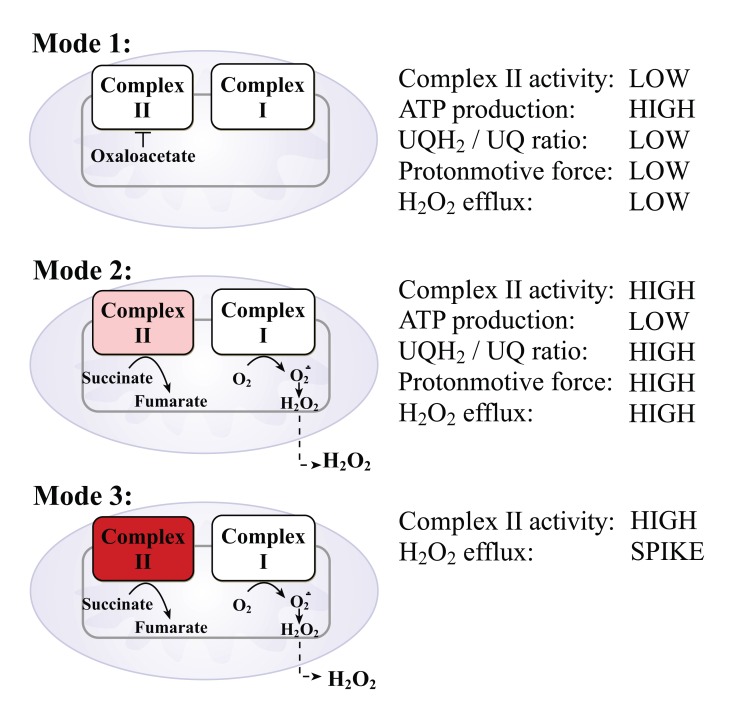
Modes of operations of mitochondrial complex II that lead to H_2_O_2_ production.

## ABBREVIATIONS

AD: = Alzheimer’s disease
ADP: = Adenosine diphosphate
ATP: = Adenosine triphosphate
DPI: = Diphenyleneiodonium
FCCP: = Carbonyl cyanide-p-trifluoromethoxy-phenylhydrazone
Grx: = Glutaredoxin
H_2_O_2_: = Hydrogen peroxide
ROS: = Reactive oxygen species
PTP1B: = Protein tyrosine phosphatase 1B
Mn-SOD: = Mn-superoxide dismutase
NAC: = N-acetylcysteine
NADPH: = Nicotinamide adenine dinucleotide phos-phate
Prx: = Peroxiredoxin
PPARγ: = Peroxisome proliferator-activated receptor-gamma agonist
RET: = Reverse electron transport
UQH_2_: = Ubiquinol
UQ: = Ubiquinone

## References

[1] Goldstein BJ, Mahadev K, Wu X (2005). Redox paradox: insulin action is facilitated by insulin-stimulated reactive oxygen species with multiple potential signaling targets. Diabetes.

[2] Mukherjee SP, Lane RH, Lynn WS (1978). Endogenous hydrogen peroxide and peroxidative metabolism in adipocytes in response to insulin and sulfhydryl reagents. Biochem Pharmacol.

[3] May JM, de Haen C (1979). Insulin-stimulated intracellular hydrogen peroxide production in rat epididymal fat cells. J. Biol Chem.

[4] Krieger-Brauer HI, Kather H (1992). Human fat cells possess a plasma membrane-bound H_2_O_2_-generating system that is activated by insulin via a mechanism bypassing the receptor kinase. J Clin Invest.

[5] Krieger-Brauer HI, Medda P K, Kather H (1997). Insulin-induced activation of NADPH-dependent H_2_O_2_ generation in human adipocytes plasma membranes is mediated by Gαi2. J Biol Chem.

[6] Mahadev K, Motoshima H, Wu X, Ruddy JM, Arnold RS, Cheng G, Lambeth JD, Goldstein BJ (2004). The NAD(P)H oxidase homolog Nox4 modulates insulin-stimulated generation of H_2_O_2_ and plays an integral role in insulin signal transduction. Mol Cell Biol.

[7] Mahadev K, Wu X, Zilbering A, Zhu L, Lawrence JTR, Goldstein BJ (2001). Hydrogen peroxide generated during cellular insulin stimulation is integral to activation of the distal insulin signaling cascade in 3T3-L1 adipocytes. J Biol Chem.

[8] Mahadev K, Zilbering A, Zhu L, Goldstein BJ (2001). Insulin-stimulated hydrogen peroxide reversibly inhibits protein-tyrosine phosphatase 1B *in vivo* and enhances the early insulin action cascade. J Biol Chem.

[9] Galic S, Hauser C,  Kahn BB, Haj FG, Neel BG, Tonks NK, Tiganis T (2005). Coordinated regulation of insulin signaling by the protein tyrosine phosphatases PTP1B and TCPTP. Mol Cell Biol.

[10] Banks WA (2004). The source of cerebral insulin. Eur J Pharmacol.

[11] White MF, Kahn CR (1994). The insulin signaling system. J Biol Chem.

[12] Accili D, Drago J, Lee EJ, Johnson MD, Cool MH, Salvatore P, Asico LD, Jose PA, Taylor SI, Westphal H (1996). Early neonatal death in mice homozygous for a null allele of the insulin receptor gene. Nat Genet.

[13] Joshi RL, Lamothe B, Cordonnier N, Mesbah K, Monthioux E, Jami J, Bucchini D (1996). Targeted disruption of the insulin receptor gene in the mouse results in neonatal lethality. EMBO J.

[14] Hatfield JS, Millard WJ, Smith CJ (1974). Short-term influence of intra-ventromedial hypothalamic administration of insulin on feeding in normal and diabetic rats. Pharmacol Biochem. Behav.

[15] Brief DJ, Davis JD (1984). Reduction of food intake and body weight by chronic intraventricular insulin infusion. Brain Res Bull.

[16] Brüning JC, Gautam D, Burks DJ, Gillette J, Schubert M, Orban PC, Klein R, Krone W, Müller-Wieland D, Kahn CR (2000). Role of brain insulin receptor in control of body weight and reproduction. Science.

[17] Hirvonen J, Virtanen KA, Nummenmaa L, Hannukainen JC, Honka MJ, Bucci M, Nesterov SV, Parkkola R, Rinne J, Iozzo P, Nuutila P (2011). Effects of insulin on brain glucose metabolism in impaired glucose tolerance. Diabetes.

[18] Zhao WQ, Chen H, Quon MJ, Alkon DL (2004). Insulin and the insulin receptor in experimental models of learning and memory. Eur J Pharmacol.

[19] Dou JT, Chen M, Dufour F, Alkon DL, Zhao WQ (2005). Insulin receptor signaling in long-term memory consolidation following spatial learning. Learn Mem.

[20] Niswender KD, Morrison CD, Clegg DJ, Olson R, Baskin DG, Myers MG Jr, Seeley RJ, Schwartz MW (2003). Insulin activation of phosphatidylinositol 3-kinase in the hypothalamic arcuate nucleus: a key mediator of insulin-induced anorexia. Diabetes.

[21] Goldstein BJ, Dudley AL (1990). The rat insulin receptor: primary structure and conservation of tissue-specific alternative messenger RNA splicing. Mol Endocrinol.

[22] Mosthaf L, Grako K, Dull TJ, Coussens L, Ullrich A, McClain DA (1990). Functionally distinct insulin receptors generated by tissue-specific alternative splicing. EMBO J.

[23] Gammeltoft S, Fehlmann M, Van Obberghen E (1985). Insulin receptors in the mammalian central nervous system: binding characteristics and subunit structure. Biochimie.

[24] Yamaguchi Y, Flier JS, Yokota A, Benecke H, Backer JM, Moller DE (1991). Functional properties of two naturally occurring isoforms of the human insulin receptor in Chinese hamster ovary cells. Endocrinology.

[25] Frasca F, Pandini G, Scalia P, Sciacca L, Mineo R, Costantino A, Goldfine ID, Belfiore A, Vigneri R (1999). Insulin receptor isoform A a newly recognized, high-affinity insulin-like growth factor II receptor in fetal and cancer cells. Mol Cell Biol.

[26] Denley A, Bonython ER, Booker GW, Cosgrove LJ, Forbes BE, Ward CW, Wallace JC (2004). Structural determinants for high-affinity binding of insulin-like growth factor II to insulin receptor (IR)-A, the exon 11 minus isoform of the IR. Mol Endocrinol.

[27] Havrankova J, Roth J, Brownstein M (1978). Insulin receptors are widely distributed in the central nervous system of the rat. Nature.

[28] Abbott MA, Wells DG, Fallon JR (1999). The insulin receptor tyrosine kinase substrate p58/53 and the insulin receptor are components of CNS synapses. J Neurosci.

[29] Unger J, McNeill TH, Moxley RT 3rd, White M, Moss A, Livingston JN (1989). Distribution of insulin receptor-like immunoreactivity in the rat forebrain. Neuroscience.

[30] Rosen OM, Herrera R, Olowe Y, Petruzzelli LM, Cobb MH (1983). Phosphorylation activates the insulin receptor tyrosine protein kinase. Proc Natl Acad Sci USA.

[31] Tornqvist HE, Pierce MW, Frackelton AR, Nemenoff RA, Avruch J (1987). Identification of insulin receptor tyrosine residues autophosphorylated *in vitro*. J Biol Chem.

[32] White MF, Shoelson SE, Keutmann H, Kahn CR (1988). A cascade of tyrosine autophosphorylation in the β-subunit activates the phosphotransferase of the insulin receptor. J Biol Chem.

[33] Wälchli S, Curchod ML, Gobert RP, Arkinstall S, Hooft van Huijsduijnen R (2000). Identification of tyrosine phosphatases that dephosphorylate the insulin receptor. A brute force approach based on "substrate-trapping" mutants. J Biol Chem.

[34] Byon JC, Kusari AB, Kusari J (1998). Protein-tyrosine phosphatase-1B acts as a negative regulator of insulin signal transduction. Mol Cell Biochem.

[35] Goldstein BJ, Bittner-Kowalczyk A, White MF, Harbeck M (2000). Tyrosine dephosphorylation and deactivation of insulin receptor substrate-1 by protein-tyrosine phosphatase 1B: possible facilitation by the formation of a ternary complex with the Grb2 adaptor protein. J Biol Chem.

[36] Picardi PK, Calegari VC, Prada Pde O, Moraes JC, Araújo E, Marcondes MC, Ueno M, Carvalheira JB, Velloso LA, Saad MJ (2008). Reduction of hypothalamic protein tyrosine phosphatase improves insulin and leptin resistance in diet-induced obese rats. Endocrinology.

[37] Rajala RV, Tanito M, Neel BG, Rajala A (2010). Enhanced retinal insulin receptor-activated neuroprotective survival signal in mice lacking the protein-tyrosine phosphatase-1B gene. J Biol Chem.

[38] Elchebly M, Payette P, Michaliszyn E, Cromlish W, Collins S, Loy AL, Normandin D, Cheng A, Himms-Hagen J, Chan CC, Ramachandran C, Gresser MJ, Tremblay ML, Kennedy BP (1999). Increased insulin sensitivity and obesity resistance in mice lacking the protein tyrosine phosphatase-1B gene. Science.

[39] Klaman LD, Boss O, Peroni OD, Kim JK, Martino JL, Zabolotny JM, Moghal N, Lubkin M, Kim YB, Sharpe AH, Stricker-Krongrad A, Shulman GI, Neel BG, Kahn BB (2000). Increased energy expenditure, decreased adiposity, and tissue-specific insulin sensitivity in protein-tyrosine phosphatase 1B-deficient mice. Mol Cell Biol.

[40] Denu JM, Tanner KG (1998). Specific and reversible inactivation of protein tyrosine phosphatases by hydrogen peroxide: evidence for a sulfenic acid intermediate and implications for redox regulation. Biochemistry.

[41] Barrett WC, DeGnore JP, Keng YF, Zhang ZY, Yim MB, Chock PB (1999). Roles of superoxide radical anion in signal transduction mediated by reversible regulation of protein-tyrosine phosphatase 1B. J Biol Chem.

[42] Lee SR, Kwon KS, Kim SR, Rhee SG (1998). Reversible inactivation of protein-tyrosine phosphatase 1B in A431 cells stimulated with epidermal growth factor. J Biol Chem.

[43] Barrett WC, DeGnore JP, König S, Fales HM, Keng YF, Zhang ZY, Yim MB, Chock PB (1999). Regulation of PTP1B *via* glutathionylation of the active site cysteine 215. Biochemistry.

[44] van Montfort RL, Congreve M, Tisi D, Carr R, Jhoti H (2003). Oxidation state of the active-site cysteine in protein tyrosine phosphatase 1B. Nature.

[45] Salmeen A, Andersen JN, Myers MP, Meng TC, Hinks JA, Tonks NK, Barford D (2003). Redox regulation of protein tyrosine phosphatase 1B involves a sulphenyl-amide intermediate. Nature.

[46] Hwang JJ, Lim JH, Kwon JH, Lee KY, Hur KC (1995). Effect of nerve growth factor, insulin, and extracellular matrix proteins on the neurite outgrowth of SK-N-BE(2) human neuroblastoma cells. Mol Cells.

[47] Seo JH, Ahn Y, Lee SR, Yeol Yeo C, Chung Hur K (2005). The major target of the endogenously generated reactive oxygen species in response to insulin stimulation is phosphatase and tensin homolog and not phosphoinositide-3 kinase (PI-3 kinase) in the PI-3 kinase/Akt pathway. Mol Biol Cell.

[48] Hwang J-J, Hur KC (2005). Insulin cannot activate extracellular-signalrelated kinase due to inability to generate reactive oxygen species in SK-N-BE(2) human neuroblastoma cells. Mol Cells.

[49] Jaillard T, Roger M, Galinier A, Guillou P, Benani A, Leloup C, Casteilla L, Pénicaud L, Lorsignol A (2009). Hypothalamic reactive oxygen species are required for insulin-induced food intake inhibition: an NADPH oxidase-dependent mechanism. Diabetes.

[50] Majander A, Finel M, Wikstrom M (1994). Diphenyleneiodonium inhibits reduction of iron-sulfur clusters in the mitochondrial NADH-ubiquinone oxidoreductase (Complex I). J Biol Chem.

[51] Li Y, Trush MA (1998). Diphenyleneiodonium, an NAD(P)H oxidase inhibitor also potently inhibits mitochondrial reactive oxygen species production. Biochem Biophys Res Commun.

[52] Liu Y, Fiskum G, Schubert D (2002). Generation of reactive oxygen species by the mitochondrial electron transport chain. J Neurochem.

[53] Storozhevykh TP, Senilova YE, Persiyantseva NA, Pinelis VG, Pomytkin IA (2007). Mitochondrial respiratory chain is involved in insulin-stimulated hydrogen peroxide production and plays an integral role in insulin receptor autophosphorylation in neurons. BMC Neuroscience, [Online].

[54] Chance B, Sies H, Boveris A (1979). Hydroperoxide metabolism in mammalian organs. Physiol Rev.

[55] Murphy MP (2009). How mitochondria produce reactive oxygen species. Biochem J.

[56] Votyakova TV, Reynolds IJ (2001). DeltaPsi(m)-Dependent and -independent production of reactive oxygen species by rat brain mitochondria. J Neurochem.

[57] Kudin AP, Bimpong-Buta NY, Vielhaber S, Elger CE, Kunz WS (2004). Characterization of superoxide-producing sites in isolated brain mitochondria. J Biol Chem.

[58] Han D, Canali R, Rettori D, Kaplowitz N (2003). Effect of glutathione depletion on sites and topology of superoxide and hydrogen peroxide production in mitochondria. Mol Pharmacol.

[59] Muller FL, Liu Y, Abdul-Ghani MA, Lustgarten MS, Bhattacharya A, Jang YC, Van Remmen H (2008). High rates of superoxide production in skeletal-muscle mitochondria respiring on both complex I- and complex II-linked substrates. Biochem J.

[60] Fridovich I (1997). Superoxide anion radical (O2-.), superoxide dismutases, and related matters. J Biol Chem.

[61] Okado-Matsumoto A, Fridovich I (2001). Subcellular distribution of superoxide dismutases (SOD) in rat liver. J Biol Chem.

[62] Fridovich I (1995). Superoxide radical and superoxide dismutases. Annu Rev Biochem.

[63] Oberley LW (2005). Mechanism of the tumor suppressive effect of MnSOD overexpression. Biomed Pharmacother.

[64] Cino M, Del Maestro RF (1989). Generation of hydrogen peroxide by brain mitochondria: the effect of reoxygenation following postdecapitative ischemia. Arch Biochem Biophys.

[65] Korshunov SS, Skulachev VP, Starkov AA (1997). High protonic potential actuates a mechanism of production of reactive oxygen species in mitochondria. FEBS Lett.

[66] Starkov AA, Fiskum G (2003). Regulation of brain mitochondrial H_2_O_2_ production by membrane potential and NAD(P)H redox state. J Neurochem.

[67] Korshunov SS, Korkina OV, Ruuge EK, Skulachev VP, Starkov AA (1998). Fatty acids as natural uncouplers preventing generation of O2.- and H_2_O_2_ by mitochondria in the resting state. FEBS Lett.

[68] Gutman M, Kearney EB, Singer TP (1971). Control of succinate dehydrogenase in mitochondria. Biochemistry.

[69] Treberg JR, Quinlan CL, Brand MD (2011). Evidence for two sites of superoxide production by mitochondrial NADH-ubiquinone oxidoreductase (complex I). J Biol Chem.

[70] Zoccarato F, Cavallini L, Alexandre A (2009). Succinate is the controller of O2-/H_2_O_2_ release at mitochondrial complex I : negative modulation by malate, positive by cyanide. J Bioenerg Biomembr.

[71] Pomytkin IA, Kolesova OE (2003). Effect of insulin on the rate of hydrogen peroxide generation in mitochondria. Bull Exp Biol Med.

[72] Wojtczak L, Wojtczak AB, Ernster L (1969). The inhibition of succinate dehydrogenase by oxalacetate. Biochim Biophys Acta.

[73] Ackrell BA, Kearney EB, Edmondson D (1975). Mechanism of the reductive activation of succinate dehydrogenase. J Biol Chem.

[74] Ackrell BA, Kearney EB, Mayr M (1974). Role of oxalacetate in the regulation of mammalian succinate dehydrogenase. J Biol Chem.

[75] Gutman M, Kearney EB, Singer TP (1971). Multiple control mechanisms for succinate dehydrogenase in mitochondria. Biochem Biophys Res Commun.

[76] Gutman M, Kearney EB, Singer TP (1971). Regulation of succinate dehydrogenase activity by reduced coenzymes Q10. Biochemistry.

[77] Nulton-Persson AC, Szweda LI (2001). Modulation of mitochondrial function by hydrogen peroxide. J Biol Chem.

[78] Moser MD, Matsuzaki S, Humphries KM (2009). Inhibition of succinate-linked respiration and complex II activity by hydrogen peroxide. Arch Biochem Biophys.

[79] Tretter L, Adam-Vizi V (2000). Inhibition of Krebs cycle enzymes by hydrogen peroxide: A key role of [alpha]-ketoglutarate dehydrogenase in limiting NADH production under oxidative stress. J Neurosci.

[80] Pomytkin IA, Kolesova OE (2002). Key role of succinate dehydrogenase in insulin-induced inactivation of protein tyrosine phosphatases. Bull Exp Biol Med.

[81] Komaromy-Hiller G, Sundquist PD, Jacobsen LJ, Nuttall KL (1997). Serum succinate by capillary zone electrophoresis: marker candidate for hypoxia. Ann Clin Lab Sci.

[82] Hochachka PW, Dressendorfer RH (1976). Succinate accumulation in man during exercise. Eur J Appl Physiol Occup Physiol.

[83] Bessman SP, Mohan C, Zaidise I (1986). Intracellular site of insulin action: mitochondrial Krebs cycle. Proc Natl Acad Sci USA.

[84] Navarro A, Boveris A (2007). The mitochondrial energy transduction system and the aging process. Am J Physiol Cell Physiol.

[85] LaFrance R, Brustovetsky N, Sherburne C, Delong D, Dubinsky JM (2005). Age-related changes in regional brain mitochondria from Fischer 344 rats. Aging Cell.

[86] Xiong J, Verkhratsky A, Toescu EC (2002). Changes in mitochondrial status associated with altered Ca2+ homeostasis in aged cerebellar granule neurons in brain slices. J Neurosci.

[87] Santos RX, Correia SC, Wang X, Perry G, Smith MA, Moreira PI, Zhu X (2010). Alzheimer's disease: diverse aspects of mitochondrial malfunctioning. Int J Clin Exp Pathol.

[88] Moreira PI, Duarte AI, Santos MS, Rego AC, Oliveira CR (2009). An integrative view of the role of oxidative stress mitochondria and insulin in Alzheimer's disease. Alzheimers Dis.

[89] Swerdlow RH, Khan SM (2009). The Alzheimer's disease mitochondrial cascade hypothesis: an update. Exp Neurol.

[90] Sies H (1991). Oxidative stress: from basic research to clinical application. Am J Med.

[91] Fatokun AA, Stone TW, Smith RA (2007). Cell death in rat cerebellar granule neurons induced by hydrogen peroxide *in vitro*: mechanisms and protection by adenosine receptor ligands. Brain Res.

[92] Halliwell B, Gutteridge JM (1990). Role of free radicals and catalytic metal ions in human disease: an overview. Methods Enzymol.

[93] Drechsel DA, Patel M (2010). Respiration-dependent H_2_O_2_ removal in brain mitochondria *via* the thioredoxin/peroxiredoxin system. J Biol Chem.

[94] Erikson KM, Pinero DJ, Connor JR, Beard JL (1997). Regional brain iron ferritin and transferrin concentrations during iron deficiency and iron repletion in developing rats. J Nutr.

[95] Park S, You X, Imlay JA (2005). Substantial DNA damage from submicromolar intracellular hydrogen peroxide detected in Hpx- mutants of *Escherichia coli*. Proc Natl Acad Sci USA.

[96] Gardner CD, Eguchi S, Reynolds CM, Eguchi K, Frank GD, Motley ED (2003). Hydrogen peroxide inhibits insulin signaling in vascular smooth muscle cells. Exp Biol Med (Maywood).

[97] Hansen LL, Ikeda Y, Olsen GS, Busch AK, Mosthaf L (1999). Insulin signaling is inhibited by micromolar concentrations of H_2_O_2_. Evidence for a role of H_2_O_2_ in tumor necrosis factor alpha-mediated insulin resistance. J Biol Chem.

[98] Cox AG, Winterbourn CC, Hampton MB (2009). Mitochondrial peroxiredoxin involvement in antioxidant defence and redox signalling. Biochem J.

[99] Adimora NJ, Jones DP, Kemp ML (2010). A model of redox kinetics implicates the thiol proteome in cellular hydrogen peroxide responses. Antioxid Redox Signal.

[100] Peskin AV, Low FM, Paton LN, Maghzal GJ, Hampton MB, Winterbourn CC (2007). The high reactivity of peroxiredoxin 2 with H_2_O_2_ is not reflected in its reaction with other oxidants and thiol reagents. J Biol Chem.

[101] Kletzien RF, Harris PK, Foellmi LA (1994). Glucose-6-phosphate dehydrogenase: a "housekeeping" enzyme subject to tissue-specific regulation by hormones nutrients and oxidant stress. FASEB J.

[102] Regino CAS, Richardson DE (2007). Bicarbonate-catalyzed hydrogen peroxide oxidation of cysteine and related thiols. Inorganica Chimica Acta.

[103] Chiu DT, Stults FH, Tappel AL (1976). Purification and properties of rat lung soluble glutathione peroxidase. Biochim Biophys Acta.

[104] McClung JP, Roneker CA, Mu W, Lisk DJ, Langlais P, Liu F, Lei XG (2004). Development of insulin resistance and obesity in mice overexpressing cellular glutathione peroxidase. Proc Natl Acad Sci USA.

[105] Loh K, Deng H, Fukushima A, Cai X, Boivin B, Galic S, Bruce C, Shields BJ, Skiba B, Ooms LM,  Stepto N, Wu B, Mitchell CA, Tonks NK, Watt MJ, Febbraio MA, Crack PJ,  Andrikopoulos S, Tiganis T (2009). Reactive oxygen species enhance insulin sensitivity. Cell Metab.

[106] Craft S (2005). Insulin resistance syndrome and Alzheimer's disease: age- and obesity-related effects on memory, amyloid, and inflammation. Neurobiol Aging.

[107] Frolich L, Blum-Degen D, Bernstein HG, Engelsberger S, Humrich J, Laufer S, Muschner D, Thalheimer A, Turk A, Hoyer S, Zochling R, Boissl KW, Jellinger K, Riederer P (1998). Brain insulin and insulin receptors in aging and sporadic Alzheimer's disease. J Neural Transm.

[108] Hoyer S (2004). Glucose metabolism and insulin receptor signal transduction in Alzheimer disease. Eur J Pharmacol.

[109] de la Monte SM (2012). Therapeutic targets of brain insulin resistance in sporadic Alzheimer's disease. Front Biosci.

[110] Zhu X, Raina AK, Lee HG, Casadesus G, Smith MA, Perry G (2004). Oxidative stress signalling in Alzheimer's disease. Brain Res.

[111] Moreira PI, Zhu X, Liu Q, Honda K, Siedlak SL, Harris PL, Smith MA, Perry G (2006). Compensatory responses induced by oxidative stress in Alzheimer disease. Biol Res.

[112] Bonda DJ, Wang X, Perry G, Nunomura A, Tabaton M, Zhu X, Smith MA (2010). Oxidative stress in Alzheimer disease: a possibility for prevention. Neuropharmacology.

[113] Martins RN, Harper CG, Stokes GB, Masters CL (1986). Increased cerebral glucose-6-phosphate dehydrogenase activity in Alzheimer's disease may reflect oxidative stress. J Neurochem.

[114] Palmer AM (1999). The activity of the pentose phosphate pathway is increased in response to oxidative stress in Alzheimer's disease. J Neural Transm.

[115] Russell RL, Siedlak SL, Raina AK, Bautista JM, Smith MA, Perry G (1999). Increased neuronal glucose-6-phosphate dehydrogenase and sulfhydryl levels indicate reductive compensation to oxidative stress in Alzheimer disease. Arch Biochem Biophys.

[116] Aksenov MY, Tucker HM, Nair P, Aksenova MV, Butterfield DA, Estus S, Markesbery WR (1998). The expression of key oxidative stress-handling genes in different brain regions in Alzheimer’s disease. J Mol Neurosci.

[117] Aksenov MY, Markesbery WR (2001). Changes in thiol content and expression of glutathione redox system genes in the hippocampus and cerebellum in Alzheimer's disease. Neurosci Lett.

[118] Kim SH, Fountoulakis M, Cairns N, Lubec G (2001). Protein levels of human peroxiredoxin subtypes in brains of patients with Alzheimer's disease and Down syndrome. J Neural Transm Suppl.

[119] Cumming RC, Dargusch R, Fischer WH, Schubert D (2007). Increase in expression levels and resistance to sulfhydryl oxidation of peroxiredoxin isoforms in amyloid beta-resistant nerve cells. J Biol Chem.

[120] Krapfenbauer K, Engidawork E, Cairns N, Fountoulakis M, Lubec G (2003). Aberrant expression of peroxiredoxin subtypes in neurodegenerative disorders. Brain Res.

[121] Sultana R, Boyd-Kimball D, Cai J, Pierce WM, Klein JB, Merchant M, Butterfield DA (2007). Proteomics analysis of the Alzheimer's disease hippocampal proteome. J Alzheimers Dis.

[122] Power JH, Asad S, Chataway TK, Chegini F, Manavis J, Temlett JA, Jensen PH, Blumbergs PC, Gai WP (2008). Peroxiredoxin 6 in human brain: molecular forms, cellular distribution and association with Alzheimer's disease pathology. Acta Neuropathol.

[123] Roses AD, Saunders AM, Huang Y, Strum J, Weisgraber KH, Mahley RW (2007). Complex disease-associated pharmacogenetics: drug efficacy drug safety and confirmation of a pathogenetic hypothesis (Alzheimer's disease). Pharmacogenom J.

[124] Strum JC, Shehee R, Virley D, Richardson J, Mattie M, Selley P, Ghosh S, Nock C, Saunders A, Roses A (2007). Rosiglitazone induces mitochondrial biogenesis in mouse brain. J Alzheimers Dis.

[125] Pedersen WA, Flynn ER (2004). Insulin resistance contributes to aberrant stress responses in the Tg2576 mouse model of Alzheimer's disease. Neurobiol Dis.

[126] Watson GS, Cholerton BA, Reger MA, Baker LD, Plymate SR, Asthana S, Fishel MA, Kulstad JJ, Green PS, Cook DG, Kahn SE, Keeling ML, Craft S (2005). Preserved cognition in patients with early Alzheimer disease and amnestic mild cognitive impairment during treatment with rosiglitazone: a preliminary study. Am J Geriatr Psychiatry.

[127] Risner ME, Saunders AM, Altman JF, Ormandy GC, Craft S, Foley IM, Zvartau-Hind ME, Hosford DA, Roses AD (2006). Rosiglitazone in Alzheimer's Disease Study Group. Efficacy of rosiglitazone in a genetically defined population with mild-to-moderate Alzheimer's disease. Pharmacogenom J.

